# Supplementary material for: The Systems Biology Research Tool: evolvable open-source software
Source: BMC Syst Biol. 2008 Jun 29;2:55. doi: 10.1186/1752-0509-2-55 (PMC2446383; doi:10.1186/1752-0509-2-55)
Supplement: Additional file 1 — SBRT Archive. An archive of the current version of the Systems Biology Research Tool. [file 1752-0509-2-55-S1.zip › sbrt-1.4.0/doc/developers_guide/api/sbrt/kernel/fba/proc/InitialPointGenerator.html]

InitialPointGenerator


|  |  |  |
| --- | --- | --- |
| **Overview** \| **Package** \| **Class** \| **Use** \| **Tree** \| **Deprecated** \| **Index** \| **Help** | |  |
| **PREV CLASS**   **NEXT CLASS** | **FRAMES**    **NO FRAMES**     **All Classes** |  |
| SUMMARY: NESTED \| FIELD \| CONSTR \| METHOD | DETAIL: FIELD \| CONSTR \| METHOD |  |


---


## sbrt.kernel.fba.proc Class InitialPointGenerator

```
java.lang.Object
  sbrt.kernel.fba.proc.FbaProcess<InitialPointComputer.Results>
      sbrt.kernel.fba.proc.InitialPointGenerator
```

**All Implemented Interfaces:**: KernelProcess<InitialPointComputer.Results>

---

``` public final class InitialPointGenerator extends FbaProcess<InitialPointComputer.Results> ```

This class is used to perform initial point experiments. Initial points are
required for random flux vector experiments.

**Author:**
:   This class was written and documented by Jeremiah Wright
    while in the Wagner lab.

**See Also:**: `CdHarFluxVectorProcess`

---

**Constructor Summary**

`InitialPointGenerator(InitialPointComputer computer)`
  
          Constructs a new initial point experiment.


| **Method Summary** | |
| --- | --- |
| `Fluxome` | `getFluxome()`             Returns the fluxome used in this initial point experiment. |
| `void` | `run(ResultsManager<InitialPointComputer.Results> resultsManager)`             Generates an initial point and adds it to the provided results manager. |

**Methods inherited from class sbrt.kernel.fba.proc.FbaProcess**

`toString`

**Methods inherited from class java.lang.Object**

`clone, equals, finalize, getClass, hashCode, notify, notifyAll, wait, wait, wait`

**Constructor Detail**

### InitialPointGenerator

```
public InitialPointGenerator(InitialPointComputer computer)
```

:   Constructs a new initial point experiment.

    **Parameters:**: `computer` - the computer with which initial points will be computed.


**Method Detail**

### getFluxome

```
public Fluxome getFluxome()
```

:   Returns the fluxome used in this initial point experiment.

    :   **Specified by:**: `getFluxome` in class `FbaProcess<InitialPointComputer.Results>`
    :   **Returns:**: the fluxome used in this initial point experiment.

---


### run

```
public void run(ResultsManager<InitialPointComputer.Results> resultsManager)
```

:   Generates an initial point and adds it to the provided results manager.

    :   **Parameters:**: `resultsManager` - the results manager to which the initial point will be added.


---


|  |  |  |
| --- | --- | --- |
| **Overview** \| **Package** \| **Class** \| **Use** \| **Tree** \| **Deprecated** \| **Index** \| **Help** | |  |
| **PREV CLASS**   **NEXT CLASS** | **FRAMES**    **NO FRAMES**     **All Classes** |  |
| SUMMARY: NESTED \| FIELD \| CONSTR \| METHOD | DETAIL: FIELD \| CONSTR \| METHOD |  |


---
